# Characterizing the Ion-Conductive State of the α7-Nicotinic Acetylcholine Receptor via Single-Channel Measurements and Molecular Dynamics Simulations

**DOI:** 10.1101/2025.08.15.670429

**Published:** 2025-08-19

**Authors:** Nauman Sultan, Gisela D. Cymes, Ada Chen, Bernard Brooks, Claudio Grosman, Ana Damjanovic

**Affiliations:** † Department of Physics & Astronomy, Johns Hopkins University; ‡ National Heart, Lung, and Blood Institute; National Institutes of Health; ¶ Department of Molecular and Integrative Physiology, University of Illinois at Urbana–Champaign; § Center for Biophysics and Quantitative Biology, University of Illinois at Urbana–Champaign; ‖ Neuroscience Program, University of Illinois at Urbana–Champaign; ⊥ Department of Biophysics, Johns Hopkins University

## Abstract

The α7-nicotinic acetylcholine receptor (α7-nAChR) is a cation-selective member of the superfamily of Cys-loop receptors. Ubiquitously expressed throughout the body of vertebrate animals, this pentameric ligand-gated ion channel participates in a wide range of physiological phenomena — as diverse as synaptic transmission and the control of excessive inflammation — and is an attractive therapeutic target for novel ligands. Although notable efforts have been made to understand this receptor-channel in terms of function and structure, many questions remain unanswered despite the molecular simplicity of its homomeric assembly. Recent cryo-EM studies have provided atomic models of this channel in different conformations, thus enabling the application of atomistic molecular dynamics (MD) simulations to the study of cation conduction. We perform both single-channel patch-clamp recordings and MD simulations on the α7-nAChR. MD simulations of an α7-nAChR model (PDB ID 7KOX) reproduced the measured single-channel conductance and revealed Poissonian ion permeation, which we further modelled as a double-Poisson process incorporating inter-event delay times. We found that cations can enter the channel through lateral fenestrations in the extracellular domain although the probability of ions following this lateral pathway — rather than the axial one — is much lower than observed in simulations of other Cys-loop receptors. We also examined other atomic models (PDB ID 7EKT and 8V80) of the α7-nAChR proposed to represent partially open states of the channel and found them to be non-conductive. This study provides insight into how ions permeate through the pore of the α7-nAChR and offers a detailed analysis of an ion-conductive conformation likely to represent the physiological open state of this receptor-channel.

## Introduction

The nicotinic acetylcholine receptors (nAChRs) are neurotransmitter-gated ion channels that transduce the binding of extracellular acetylcholine (ACh) into the passive flow of inorganic cations into the cell. Along with homologous receptors to serotonin, γ-aminobutyric acid, glycine, and Zn^2+^, the nAChRs constitute the superfamily of Cys-loop receptors of vertebrate animals.^1–3^ These membrane receptors are pentameric assemblies of identical or homologous subunits arranged around a central pore. The extracellular domain (ECD) harbours the neurotransmitter-binding sites, the transmembrane domain (TMD) forms the transmembrane pore, and five short stretches of amino acids — one per subunit — link the two domains covalently^4^ ensuring their functional coupling.^5^

In vertebrate animals, nAChRs are formed by a variety of subunits (α1−α10,β1−β4,γ,δ,andε), α7 being one of the few that can assemble into fully functional homomers. Another unique aspect of the α7-nAChR is its expression in many different cell types of the animal body, which underlies its participation in a wide range of physiological phenomena and pathological conditions.

All neurotransmitter-gated ion channels (whether from the superfamily of Cys-loop receptors or not) can interconvert among a variety of conformations that differ in their ability to conduct ions and the affinity with which they bind neurotransmitters.^6^ The conformational space accessible to these receptor-channels is typically complex, but it minimally consists of three end states denoted as “closed”, “open” and “desensitized”. The open state conducts ions, whereas the closed and desensitized states do not. On the other hand, the open and desensitized states bind neurotransmitter with a higher affinity than the closed state does.^6^ In the absence of neurotransmitter, the closed state is the most stable conformation, and its occupancy approaches unity. However, because of their different neurotransmitter-binding affinities, the presence of neurotransmitter favours the open and desensitized states. Indeed, in the presence of saturating concentrations of neurotransmitter, the equilibrium occupancy of the closed state drops to nearly zero. Since the neurotransmitter-bound desensitized state is much more stable than the neurotransmitter-bound open state, the occupancy of the latter at equilibrium is, also, typically very low. As a result, the neurotransmitter-bound open state is only transiently occupied as the concentration of neurotransmitter is stepped from zero to saturating. It follows then that, in the absence of neurotransmitter, nearly all channels are closed, whereas in the presence of saturating concentrations of neurotransmitter, nearly all channels are desensitized.

In recent years, largely through the application of cryogenic electron microscopy (cryo-EM), the structures of all major types of Cys-loop receptors in at least one conformation have been solved. Not surprisingly, the unliganded closed state is the conformation most commonly isolated in the absence of neurotransmitter (or any other efficacious agonist that binds to the neurotransmitter-binding sites), and the agonist-bound desensitized state is the conformation most commonly observed in the presence of saturating full agonists. Different approaches have been followed to enrich the mixture of conformational states in the elusive agonist-bound open conformation, with the “plunge-freezing”, out-of-equilibrium method of Unwin and coworkers^7^ having proved to be the most successful, thus far.^8^ However, a few atomic models of Cys-loop receptors obtained under equilibrium conditions were proposed to display features consistent with the agonist-bound open conformation, even when time-resolved methods (in the, physiologically relevant, millisecond range) were not used; such is the case for the α7-nAChR, for example. A likely explanation for this unexpected (but hardly rare) finding is that the lipid-membrane mimetic used to maintain the protein in solution for cryo-EM imaging somehow fails to accurately recapitulate the properties of the plasma membrane, and thus fails to preserve the channel’s native conformational free-energy landscape. Whatever the case might be, an atomic model of the receptor’s open state affords the opportunity of studying the phenomenon of biological ion conduction using atomistic molecular dynamics (MD) simulation.

Before high-resolution structures of the α7-nAChR were available, an MD study^9^ used a modelled structure to estimate conductance based on the free-energy profile of the channel. More recently, studies have leveraged cryo-EM structures to explore the receptor’s functional states (open, closed, and desensitized). The first study focused on lipid interactions using both all-atom and coarse-grained simulations,^10^ and on the basis of a short MD simulation, reported an inward Na^+^ conductance of 44 pS. Another study^11^ reported a higher Na^+^ conductance of 120 pS, although its primary focus was on Markov state modeling of conformational transitions within the desensitized state. This work identified a conducting conformation of the desensitized state with low conductance. Additionally, a separate study by Zhuang et al.^12^ employed Markov state modeling to investigate the gating mechanism of α7-nAChR.

Here, we present simulations and an electrophysiological characterization of the open-channel state of the α7-nAChR. We performed all-atom MD simulation to gain mechanistic insight into the phenomenon of cation permeation through three atomic models of this receptor that were deemed to represent open or open-like states (PDB ID 7KOX,^13^ 7EKT,^14^ and 8V80^15^); these were obtained under conditions that slow down (but, certainly, do not prevent) channel desensitization. We ran multiple simulations and demonstrated the need for such multiple copies. Moreover, we computed the single-channel conductance tracking the pathway followed by the permeating ions. We found that one of the simulated atomic models (PDB ID 7KOX) recapitulates the experimentally estimated single-channel K^+^ conductance remarkably well, whereas, the other two (PDB ID 7EKT and 8V80) do not conduct currents at all..

### Experimental and Computational Methods

#### cDNA clones, Mutagenesis, and Heterologous Expression

Complementary DNA (cDNA) coding human α7-nAChR (UniProt accession number: P36544) in pcDNA3.1 was purchased from addgene (#62276); cDNA coding isoform 1 of human RIC-3 (accession number: Q7Z5B4)^16^ in pcDNA3.1 was provided by W. N. Green (University of Chicago, IL); and cDNA coding human NACHO (TMEM35A; accession number: Q53FP2)^17^ in pCMV6-XL5 was purchased from OriGene Technologies Inc. (#SC112910). The α7-nAChR was heterologously expressed in transiently transfected adherent HEK-293 cells grown at 37°C and 5% CO_2_ in 35-mm cell-culture dishes.. Transfections were performed using a calcium-phosphate method, and cells were incubated with the calcium-phosphate-DNA precipitate for 16–18 h after which the cell-culture medium (DMEM; Gibco) was replaced with fresh medium. The transfection mixture contained a total of 3.0 μg/dish of cDNAs coding the α7 subunit, RIC-3, and NACHO in a 1:5.5:5.5 ratio (by weight).

#### Electrophysiology

Single-channel currents were recorded in the cell-attached configuration at ~22°C using an Axopatch 200B amplifier (Molecular Devices). The output signal was low-pass filtered (900C; Frequency Devices) so that the effective bandwidth was DC–30 kHz. Currents were digitized (at 100 kHz) and analyzed using QuB 1.4 software (the MLab Edition). The bath solution was 142 mM NaCl, 5.4 mM KCl, 1.8 mM CaCl_2_, 1.7 mM MgCl_2_, and 10 mM HEPES/NaOH, at pH 7.4. The pipette solution was 110 mM KCl, 40 mM KF, 1 or 100 μM ACh, 3 μM PNU-120596 (a positive allosteric modulator that slows down desensitization^18^), 0.1% v/v DMSO, and 10 mM HEPES/KOH, pH 7.4. Patch pipettes, pulled from thick-walled borosilicate-glass capillary tubing (Sutter Instrument), had resistances of 7–10 MΩ when filled with pipette solution. Current amplitudes were estimated from the idealization of single-channel traces using the segmental K-Means (SKM) method implemented in QuB^19^ without any additional filtering. These current amplitudes were used to generate current-voltage plots, and the latter were used to estimate the single-channel conductance (as the slope of these linear plots). For display purposes, example single-channel traces were further low-pass filtered at 5 kHz.

#### Molecular Dynamics Model

The starting points for our MD simulations were atomic models PDB IDs 7KOX, 7EKT, and 8V80. CHARMM-GUI^20–26^ was used to create the protein–membrane bilayer system and add ions. The generated systems were electrically neutral, featuring periodic boundary conditions in all three dimensions and a bulk ion concentration of 150 mM KCl. The protonation states of ionizable residues were modelled on the basis of CHARMM-GUI recommendations at pH 7, which uses PROPKA^27,28^ calculations. In particular, all Asp and Glu residues were modelled as negatively charged, all Lys and Arg residues as positively charged, and all His residues were modeled as neutral. The membrane bilayer was composed of POPC lipids. Box dimensions, number of molecules, and disulfide bond details are included in table III. The minimum distance between periodic images of protein atoms, in every setup, along all three axes was approximately 30 Å. During the setup, pore water was added up to a radius of 9 Å in all cases, and the channel was vertically aligned for membrane placement by running PPM 2.0^29^ in CHARMM-GUI. The five calcium ions, present in the PDB models 7KOX and 8V80, were restrained during the MD simulation to the C-α atoms of Glu 44 using a harmonic restraint. These restraints were added to prevent the ions from moving out of their binding pockets.

#### Minimization and Equilibration

CHARMM36m^30^ was used as the force-field. The SHAKE algorithm^31^ was used to impose a holonomic constraint on bonds containing hydrogen atoms, and the SETTLE algorithm^32^ was used to constrain rigid water (TIP3) molecules.

Before the velocities were assigned to each atom, the energy of the system was minimized by using 5,000 steps of LocalEnergyMinimizer in OpenMM (the position was searched using the L-BFGS algorithm) and a combination of 2,500 steps of steepest descent and 2,500 steps of conjugate gradient in AMBER.

Particles in the minimized systems were assigned velocities from the Maxwell–Boltzmann distribution at a temperature of 30°C, and the systems were equilibrated. Equilibration proceeded as a 6-step process (for each replica in each setup), following the CHARMM-GUI protocol (see table I); at each step, the bond and dihedral restraints were gradually lifted until completely removed before the production run.

Pre-production started after running the system without restraints where all runs were in NPT setup (see table IV). The results and trajectories from minimization and equilibration steps were not included in the analysis.

Two different ensembles were used to simulate the systems: NPT and NVT setups were used in OpenMM^33^ and AMBER,^34–37^ respectively. For NPT systems, the pressure was maintained at 100 kPa using a MonteCarlo (MC) barostat coupling. Systems were coupled to a heat bath using Langevin dynamics with friction coefficient of 1 ps to maintain the temperature at 30°C. The ensemble details and software used for simulations are included in tables II and IV.

#### Production Runs

Production simulations had an external electric field (EF) applied,^38^ with strength adjusted to maintain the target membrane potential. Each production run was at least 200-ns long, with the longest run being 1 μs, and the total simulation time of all production runs adding up to ~ 4.8 μs. Details regarding production runs are found in tables II and IV. The systems were integrated at a time step of 2 fs, and the coordinates of atoms were saved every 10 ps.

### Post Simulation Analysis

Visual Molecular Dynamics (VMD),^39^ along with repositories HOLE^40^ and PMEPot Plugin^41^ were used to calculate ion densities, channel radius, and the electrostatic-potential map of each system. CPPTRAJ^42,43^ was used to calculate position densities of residues, to combine trajectories, and to calculate average structures. Python code was written using MDTraj^44^ to calculate channel conductance, RMSDs between structures, and to plot ion trajectories.

### Channel Conductance

Current through the receptor was calculated by counting the number of ions crossing the channel:^41,45^

(1) $$I_{r}≃\frac{1}{\Delta t}\sum_{i=1}^{N}q_{i}n_{i}.$$

**Figure 2:**
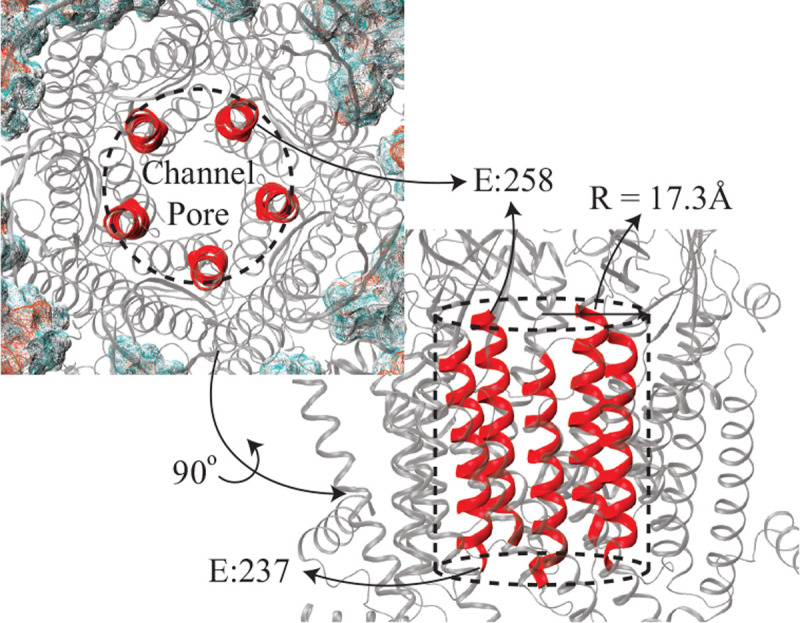
M2 helices lining the TMD pore, having Glu 237 and Glu 258 at the cytosolic and extracellular ends, respectively, are shown as red ribbons. These helices dictate the height of the cylindrical region (with radius of 17.3 Å) used for calculation of the number of ions crossing the TMD.

Here, the number of crossings, $n_{i}\in [-1,+1]$, was determined by counting ions (with charge q) that completely traverse the TMD during the simulation time ∆t. From these counts, the single-channel conductance was calculated as ($G=I_{r}/V_{e}$), where $V_{e}$ is the voltage difference ($V_{e}=L_{z}\times EF$, were $L_{z}$ is the height of the entire simulation box). The region of the TMD used to calculate ion-crossing events was defined by a right cylinder with a radius of 17.3 Å and a length determined by the z-axis coordinates of the backbone atoms of the two rings of glutamates (positions 258 and 237) at either end of the TMD (Fig. 2). A TB_ion_ (top-to-bottom, the subscript denoting the ion type) event occurs whenever an ion crosses the ring of five Glu-258 residues, enters the TMD, and exits through the ring formed by the Glu-237 residues into the ICD; this is the direction of the electric field (orthogonal to the plane of the lipid membrane). Conversely, a BT_ion_ (bottom-to-top) event occurs whenever an ion moves in the opposite direction, that is, entering from the Glu-237 ring and exiting through the Glu-258 ring. The channel radius was chosen sufficiently wide to capture the obliquity of the cylinder defined by the M2 helices of the five α7 subunits during the simulation and to ensure that an ion never escapes along this height. Given the large conductance of this receptor, only complete events were considered. That is, ions present within the TMD at the start and end of the production phase were disregarded, i.e. $n_{i}$, is either +1 or −1.

### Poisson Process to Define Events Distribution

If events are independent of one another in a disjoint time interval, and if the distribution of such events is defined by a single rate parameter λ for an interval δt, which can be translated for any time T, the distribution of events can be characterized by a homogeneous Poisson point process.

The probability density (PDF) and the cumulative distribution (CDF) functions for a Poisson process are, respectively, given by:

(2) $$f_{P}(t)=\lambda \;exp[-\lambda t],\;\text{and}$$

(3) $$F_{P}(t)=1-exp\;[-\lambda t]$$

where t is the waiting time between events — that is, the time difference between the start of consecutive events. The average number of events in a given interval can be calculated from the rate parameter as 1/λ. The waiting time $t_{i}$ for i number of events from a total of N number of events is:^46,47^

(4) $$t_{i}=-\frac{1}{\lambda }ln\left[1-\frac{i}{N}\right].$$

Equation 4 is used to generate a linear expression between the event waiting times $t_{i}$ and the distribution of events, calculated using the logarithm of ratio of remaining events ln[1−i/N]). The slope of this linear relation is the rate parameter λ, inverse of which calculates the number of events in a given time T. This method was used to assess whether the distribution of ion crossing events, as defined above, and the rate of ions crossings through the channel conform to a Poisson process.

To account for the deviation between the observed distribution of waiting times between events and the expectations of a single Poisson model, we employed a chained double-Poisson process. This model incorporates two coupled Poisson process with two independent rate parameters; $\lambda_{\text{lag}}$, depicting the lag time in events and $\lambda_{\text{cond}}$, which defines the rate of conduction. Further details of physical foundation of this method are provided in the Results section and in the Supporting Information.

The PDF and CDF of a double-Poisson distribution are expressed as:

(5) $$f_{DP}(t)=\frac{\lambda_{\text{cond}}\lambda_{\text{lag}}}{\lambda_{\text{cond}}-\lambda_{\text{lag}}}\left(exp\left[-\lambda_{\text{cond}}t\right]-exp\left[-\lambda_{\text{lag}}t\right]\right)$$

6 $$F_{DP}(t)=1-\left(\frac{exp\left[-\lambda_{\text{lag}}t\right]-exp\left[-\lambda_{\text{cond}}t\right]}{\lambda_{\text{cond}}-\lambda_{\text{lag}}}\right)$$

Derivation of these expressions are found in Supporting Information: Model of Double-Poisson Process.

## Results and Discussion

### Single-channel recordings

**Figure 3:**
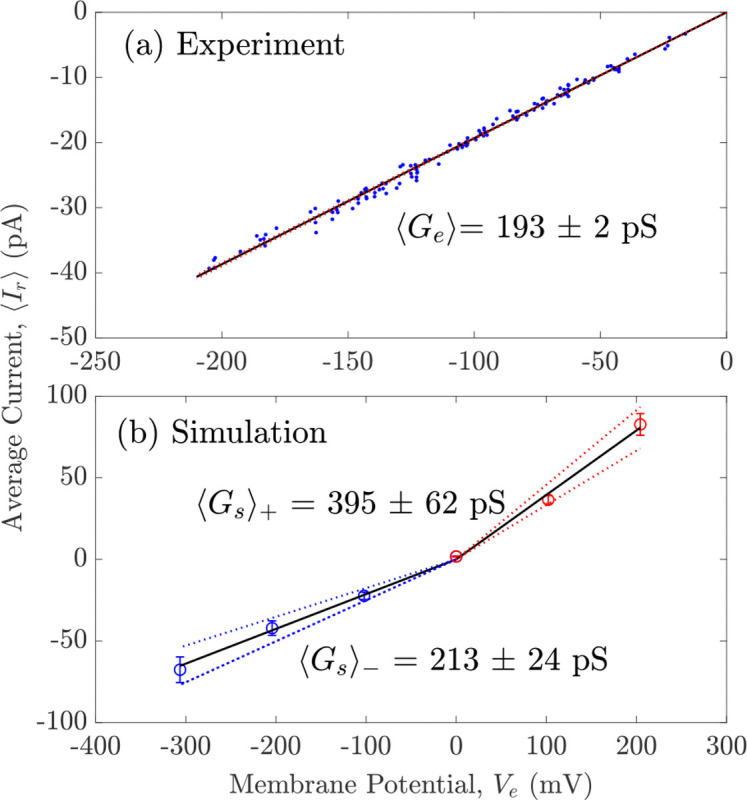
Average currents measured (a) experimentally and (b) using MD simulations at different voltages. Blue scattered points in panel (a) are current amplitudes at different membrane potentials recorded from 10 separate cell-attached patch-clamp experiments. The slope was estimated from a global fit to all the plotted points. Marked circles in (b) are average currents calculated using simulations at given voltages (error bars represent standard errors). Two solid black lines are the fitted linear current-voltage relations at negative (blue) and positive (red) potentials, separately, with upper and lower 95% confidence (dashed lines). The slopes in each case give the single-channel conductance of the channel.

**Figure 4:**
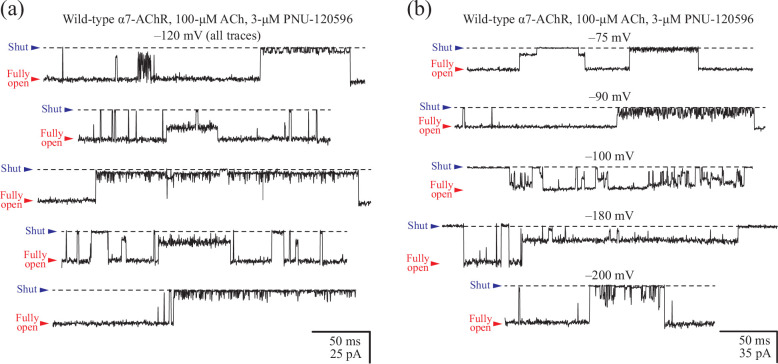
Single-channel currents from the human α7-nAChR in the presence of a positive allosteric modulator. (a, b) Single-channel activity recorded in the cell-attached configuration from the human α7-nAChR heterologously expressed in HEK-293 cells. The positive allosteric modulator PNU-120596 was present in the pipette solution at a saturating concentration (3 μM). Under these conditions, current sojourns in partially open levels of conductance were frequent and, often, very long lived. The indicated electrical potentials correspond to those applied to the pipette; openings are downward deflections; and—for display purposes—the bandwidth was narrowed to DC–5 kHz. Black dashed lines denote the zero-current baseline. The displayed current traces were recorded from different cells.

To gain a meaningful basis of comparison for our molecular simulations, we recorded single-channel currents from the (cation-selective) human wild-type α7-nAChR. To this end, we expressed this Cys-loop receptor heterologously in HEK-293 cells and performed patch-clamp recordings in the cell-attached configuration. Currents were elicited by extracellular ACh (1 or 100 μM), and openings were prolonged by PNU-120596 (3 μM), a TMD-binding ligand that slows down desensitization;^48^ in the absence of the latter, openings are exceedingly short, and thus, the single-channel conductance is prone to be underestimated. Under these conditions, and in the presence of ~155-mM K^+^ in the pipette (and the nominal absence of divalent cations), the single-channel conductance was 193 pS for inward currents (Fig. 3, top panel). This is the conductance between the fully open and the zero-current (“shut”) levels (Fig. 4). Notably, currents through the human α7-nAChR dwelled on partially open levels (Fig. 4) with a much higher probability and for much longer durations than do currents recorded through, for example, the adult-muscle AChR^49^ (a heteropentamer of $(\alpha 1{)}_{2}\beta 1\delta \varepsilon$ subunit composition). These current sublevels were difficult to characterize quantitatively because they seemed to occur essentially anywhere between the fully open and the shut levels. In other words, these sublevels could not be assigned to discrete subconductance classes. These partial openings may reflect incomplete degrees of pore expansion, protonation–deprotonation events and/or changes in the rotameric state of side chains; the reasons why they are so prominent and their amplitude is so variable in the α7-nAChR remains puzzling. Regarding the single-channel conductance of inward currents carried by K^+^, we note that the value of ~193 pS is remarkably close to the value of ~185 pS measured from the mutant adult-muscle nAChR engineered to also contain five glutamates at the intracellular end of the pore^49^ (position −1’; Glu 237 in the α7-nAChR; the wild-type muscle AChR has four glutamates and one glutamine at this position). This finding suggests that the pores of these two closely related Cys-loop receptors have very similar electrostatic properties.

### 7KOX versus 7EKT versus 8V80

**Figure 5:**
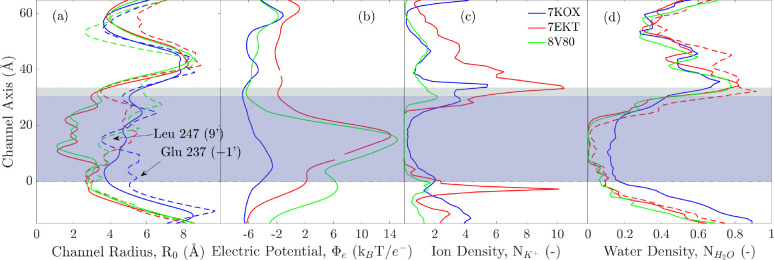
The average profiles of the three simulated structures, showing (a) solid: channel radii from MD simulations, dashed: radii from PDB structures; (b) electrostatic potentials; (c) K^+^ ions densities (normalized to density in bulk water); and (d) water count (normalized to bulk water density). The channel (vertical) axis for all structures are referenced to Glu 237.

To simulate ion conduction in the α7-nAChR, we compared MD simulations of the different structures with PDB IDs 7KOX, 7EKT, and 8V80. For each of the three atomic models, we performed three independent 200-ns long runs. No ion crossings were observed during simulations of models 7EKT and 8V80. Fig. 5 shows the pore-radii, electrostatic potentials, and the ion (K^+^) and water densities for these structures, which were calculated from the MD simulations. The relative positions of the ionizable residues along the pores of these structures from MD runs are shown in Fig. II. During the MD simulation, we observed clear hydrophobic gating in the TMD of 7EKT and 8V80 structures, with no water present in the center of the transmembrane pore, Fig. 5 (d).

The most significant difference between the conducting and non-conducting PDB models (before any energy minimization or simulations) is near Glu 237, as shown by their pore radii (dashed lines in Fig. 5 (a)). In the PDB structure of conducting model, 7KOX, the narrowest position of the PDB structure is at Leu position 247 (9’) which shifts to Glu position 237 (−1’) during MD simulation. This increase and decrease in radius at Glu 237 and Leu 247, between the average MD and PDB structures, are ~ 1 Å each. Comparing the PDB structures, at Glu 237, the pore radii of 7EKT and 8V80 are ~ 2 Å narrower than the radius of 7KOX. This narrowness may explain why water could not be placed in the pore of the channel when hydrated with CHARMM-GUI, especially at this location. Since no water molecules were placed here during the MD setup, the resulting lack of pore water pressure in the TMD of the two non-conductive structures (7EKT and 8V80) further led to the collapse of the pore, particularly after the constraints were released during the equilibration. The collapse observed during the simulations of 7EKT and 8V80 occurs at the hydrophobic center of the pore, but the narrowest region of their PDB structures (that is, again, of the atomic models without any minimization or equilibration) occurs at Glu 237. We analyzed the variation in the positions of the residues facing the pore of the TMD (Fig. III), and found that the pores are narrowest at Leu position 247 (9’). This aspect is a shared characteristic of the two non-conducting structures, which may explain why a recent MD study^11^ found that mutating hydrophobic Leu at 247 (9’) to a polar Thr (L247T) keeps the pore partially hydrated, allowing ions to pass.

To further ascertain whether the dehydration in this region is not an unintended consequence of the CHARMM-GUI hydration protocol, we attempted to place water molecules manually in the TMD of PDB IDs 7EKT and 8V80; the energy of the system for the latter could not be minimized. We also made efforts to hydrate the pore of both PDBs by constraining the backbone (to prevent their collapse) and allowed the system to equilibrate for 10 ns. The setup of the atomic model 7EKT, which did minimize, after the 10-ns of equilibration, had few molecules of water in the TMD region (dashed red curve in Fig. 5 (d)). However, on the basis of a 200-ns long simulation there still was no permeation of ions through the TMD of 7EKT.

α7-nAChR cryo-EM atomic models 7KOX, 7EKT, and 8V80 were obtained under equilibrium conditions in the presence of both high-efficacy ECD-binding agonists and TMD-binding positive allosteric modulators. The reasons behind the marked differences between these atomic models are puzzling inasmuch as they were obtained under similar conditions. Certainly, all three cases, a highly efficacious agonist was bound to the neurotransmitter-binding sites, and a ligand that prolongs the mean lifetime of the open-channel conformation (a “positive allosteric modulator”) was bound to the TMD. The different membrane mimetics used to solubilize the protein (a lipid nanodisc for 7KOX, and detergent micelles for 7EKT and 8V80) may underlie, to some degree, these different results. However, the recent report of an open-channel model of the detergent-solubilized α7-nAChR (obtained in the presence of a TMD-binding ligand but in the absence of orthosteric agonist^50^) clearly suggests that a more complex, less predictable scenario is at play, here.

To summarize, on the basis of results from our MD simulations, the 7EKT and 8V80 atomic models remain non-conductive throughout the MD runs. Note, however, that the pore-radius profiles of the atomic models and their MD-simulated counterparts differ significantly in all three cases. For example, the narrowest constriction of the transmembrane pore of 7KOX moves from the ring of Leu 247 side chains halfway through the pore (position 9’), in the model, to the intracellular mouth, around Glu 237 (position −1’), upon simulation. To date, as far as the authors are aware, the only conducting atomic model of the α7 nAChR reconstituted in a lipid bilayer is 7KOX. Therefore, we focused the rest of our MD study on the analysis of ion translocation through this model.

### Current–voltage characteristics

The obtained (single-channel) current–voltage (I-V) plots from the MD simulations are shown in Fig. 3, panel (b). The values of the membrane potential, the length of each simulation, and the number of replicas are summarized in table V. The slopes of the linear fits were used to calculate the single-channel conductance.

For inward currents, the calculated single-channel conductance of K^+^ ions (213 ± 24 pS) agrees closely with the experimentally obtained value (193 ± 2 pS). For outward currents, the calculated single-channel conductance was 395 ± 62 pS; the ratio of outward to inward currents is ~2.

We compare this ratio to that calculated in another MD study,^10^ (using Na^+^ rather than K^+^), which also showed that the outward conductance is higher, with a ratio of ~5 (~223 pS outward, and ~44 pS inward). Yet another recent all-atom MD study^11^ reported an average conductance of ~120 pS for Na^+^ ions, with an inward to outward ratio between 1.3 and 2.0.

### Channel Profile and Electrostatics

**Figure 6:**
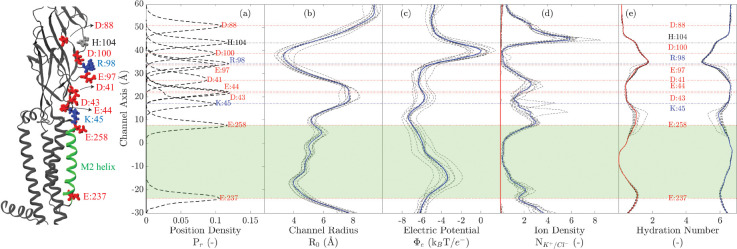
Channel profile of the α7-nAChR conducting structure (PDB ID 7KOX) during the MD simulations. For reference, a single α7 subunit is shown, on left, with positions of residues labelled. Along the vertical axis, zero is assigned to the average positions of Val 251 atoms, and the TMD (region defined between Glu 258 and Glu 237) is shaded in green. Panel (a) is the position density of ionizable residues, (b) is the variation in channel radius, (c) is the electrostatic potential map of the channel, (d) shows the density of K^+^ (in blue) and Cl^−^ ions (in red) normalized to their concentrations in bulk water. Panel (e) is the hydration number of K^+^ ions inside the channel (in blue) and the number water molecules replaced by oxygen atoms of protein residues (in red).

The positions of ionizable residues ($P_{r}$) along the pore, the channel radius ($R_{0}$), the electrostatic potential ($\Phi_{e}$), the ion densities, and the hydration number of ions along the channel pore are plotted in Fig. 6.

The position density in Fig. 6 (a) shows the normalized probability of finding ionizable residues along the inner lining of the pore (the pore axis of the channel). These were obtained by considering all atoms (including hydrogen atoms) in all five subunits. The plots are average positions (calculated considering all 5 subunits) on the basis of data recorded every 100 ps from all simulations.

The radius of the channel pore was calculated using HOLE (with default van der Waals radii of atoms) every 100 ps at vertical intervals of 0.5 Å for each simulation (Fig. 6 (b)). The average radii from individual runs are plotted as dotted lines, and the solid blue line represents the radius of the channel averaged over all runs. The narrowest constriction of this particular model occurs between Arg 98 and Asp 100, in the extracellular domain; the second narrowest constriction occurs at the intracellular end of the pore, at position Glu 237.

The electrostatic potential along the channel pore, $\Phi_{e}$, was calculated incorporating the smooth partial-mesh Ewald method (PME) using the PMEPot plugin in VMD. These calculations take into account all charges in the simulation box, which include ions and charged residues of the protein. The ion density was obtained by counting the number of ions in 0.5-Å-thick disks of radius 17.3 Å along the vertical axis of the pore. The plotted density was normalized to the density of ions in 150-mM KCl in water. The potential and number of ions were measured from frames saved every 100 ps, and the average of each run was plotted as dashed lines in Fig. 6 (c) and (d) respectively; the solid blue line represents the average over all 10 runs in each case. The individual result of each run deviates from the average, which reflects the variation in charge distribution between different runs. The highest potential barrier faced by ions moving along the longitudinal axis of the pore occurs near the narrowest constriction, in the ECD.

Fig. 6 (e) shows the hydration number, that is, the number of water molecules in the first hydration shell of K^+^ inside the channel. The threshold radius for this calculation (3.52 Å) was given by the radial density function of K^+^ created using a water-box MD simulation with the same ion concentration (150-mM) as that used in the simulations of ion permeation. The solid blue curve is the average number of water molecules in the first hydration shell, and the dotted lines show values from each simulation run. The solid red curve is the average number of water oxygen atoms replaced by oxygens from protein residues; the dotted lines show values from each run. Oxygen atoms of protein replace those of water most extensively at the narrowest constriction of the pore (residues Glu 97 and Arg 98, in the ECD), followed by the rings of glutamates at either end of the transmembrane pore (Glu 237 and Glu 258). At most, K^+^ loses two water molecules from the first hydration shell (out of an average of six) as it permeates during the MD simulation.

### Event Distribution and Statistics

The total run time of the simulations at −102 mV was 3.6 μs, and the distribution of events from all replicas is summarized in table 1. The indices of the table represent individual MD runs. Any simulation longer than 200 ns was broken down into sections of 200-ns, and these are further identified by Roman letters. For example, run 6 was 400 ns long, and 6.a and 6.b are considered independent runs of 200 ns. Event counts — that is, the first four columns of the table after indices — are either positive (${TB}_{\text{ion}}$), when an ion enters from the ECD (Glu 237) and exits into the ICD (Glu 258), or negative (${BT}_{\text{ion}}$), when an ion crosses the TMD in the opposite direction. The second-to-last column of this table gives the net number of events, defined here as the net translocation of charges through the transmembrane pore in either direction. The table reveals a significant variation in the number of net events, and hence conductance, between different MD runs, with the overall conductance ranging between 79 and 399 pS. This indicates that multiple measurements are needed to correctly estimate the mean conductance, as individual measurements could result in a large error. To estimate the variability of the individual measurements relative to the mean, we calculate the ratio of standard deviation to average number of ${TB}_{K^{+}}$, and ${BT}_{K^{+}}$ events to be 0.24 and 0.33 respectively, suggesting that the ion conduction in the TB direction is more consistent. The distribution of event durations is shown in Fig. I for ion translocations in either direction. These probability densities were fitted with a log-normal distribution (dashed lines on the figure), the mean duration of events being ~1.24 ns for both inward and outward events. We calculated the diffusion coefficient of K^+^ in the TMD pore of the channel to be 0.36 m^2^/s, that is, ~0.2 times the rate of free diffusion of this ion in water along a single dimension.^51^

To ascertain whether the differences in the number of events between different runs stem from conformational differences of the channel, we performed several types of analyses. These were: a) Computing the average structures from all 10 individual simulations performed at −102 mV and calculating the RMSD between these structures; b) Performing principal component analysis (PCA); and c) Performing t-distributed stochastic neighbour embedding (tSNE). Our results failed to identify a correlation between computed single-channel conductances and structural features. A brief overview of these analyses and results are included in the supporting information, section: Correlation Analysis between Conductance and Structural Variation.

**Figure 7:**
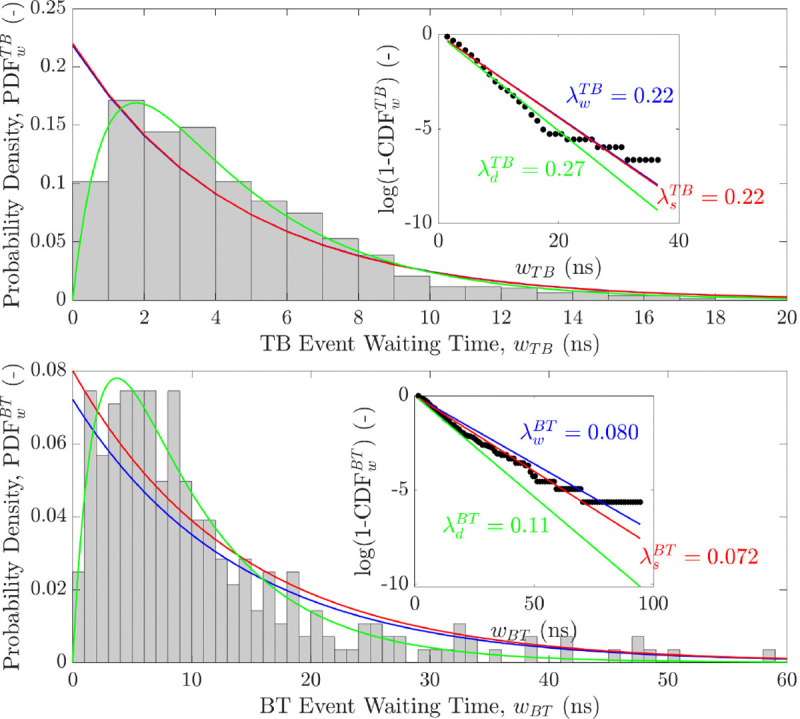
Probability density distributions of waiting times between consecutive ${TB}_{K^{+}}$ (upper panel) and ${BT}_{K^{+}}$ (lower panel) events. Data fitted to a pure Poisson (in blue, eq. 2) and double-Poisson process (in green, eq. 5) from all 10 simulations (see table 1) at $V_{e}=-102$ mV. The linear fits to the data using eq. 4 are show in inserts for each plot. The red curves are plots with the rate coefficients estimated from average number of events calculated from the simulations (see table 1). The blue curves are calculations from the linear fit. The green curves are double-Poisson process, the slope on the linear fit does not account for the lag time. The rate coefficients are as shown on the plots, for TB events these are the same (the red plot is overlapping the blue plot).

Next, we modelled the distribution of ion-crossing events with a temporal Poisson process. Fig. 7 shows the probability density function of waiting times (${PDF}_{w}$) between consecutive events. Waiting time is the time interval between the start of two consecutive events. This analysis was performed for potassium-ion crossing events only, and the distributions of crossings in either direction are shown separately.

The blue line on the plots shows an exponential decay with Poisson parameters $\lambda_{s}^{TB}=0.22{\;ns}^{-1}$ and $\lambda_{s}^{BT}=0.072{\;ns}^{-1}$ obtained by fitting the data to a Poisson distribution. The linear regressions were forced to intercept the y-axis at 0, which is the shortest possible waiting time. The solid red lines are the exponential decay with Poisson parameter calculated from the average number of events $\lambda_{s}^{TB}=44/200=0.22$ and $\lambda_{s}^{BT}=16/200=0.08$ (events/ns). The inserts of the figure show the plots of the distribution based on the expression in eq. 4; the blue and red lines are linear fits with slopes $1/\lambda_{w}$ and $1/\lambda_{s}$ respectively, both passing through the origin.

To better reconcile the discrepancy between the predictions of a pure Poisson process—which allows arbitrarily short inter-event intervals—and the observed delay between consecutive conduction events in simulations, we examined individual ion trajectories. Visual inspection suggests that potassium ions are temporarily delayed when traversing regions near the charged residues at the termini of the transmembrane domain (TMD). These charged residues are surrounded by additional potassium ions which appear to electrostatically repel incoming cations. For example, an ion initiating a top-to-bottom (TB) event near Glu-258 may transiently occupy a position that inhibits subsequent ions from entering the pore. As a result, the next ion must wait until the preceding one has moved sufficiently deeper into the TMD, introducing a delay not captured by a simple Poisson process. To model this behaviour, we employed a double-Poisson process, comprising two sequential Poisson-distributed waiting times: the first accounts for the initial lag due to inter-ion electrostatic interference, and the second models the stochastic interval between conduction events themselves.

To model the distribution of waiting times using the double-Poisson process, we optimized the rate parameters, $\lambda_{\text{lag}}$ and $\lambda_{\text{cond}}$, in eq. 5, governing this model to fit the simulation results. The resulting optimized distributions are plotted in green in Fig. 7. The lag times in this model for TB and BT events are 1 ns and 1.53 ns, respectively, while the average waiting times are 3.62 ns and 8.14 ns.

According to a pure Poisson model, the average number of ${TB}_{K^{+}}$ events is estimated to be 44, which is identical to the average number of ${TB}_{K^{+}}$ events calculated from the 200-ns long simulation. For ${BT}_{K^{+}}$ events, the average number of events estimated from the Poisson distribution is ~14.4 in 200 ns, and the average number of events calculated from simulations is 16. In contrast, the average number of events calculated from the double-Poisson model over 200 ns is 200/(1 + 3.62) ≃ 43.3 for ${TB}_{K^{+}}$ and 200/(1.53 + 8.14) ≃ 20.7 for ${BT}_{K^{+}}$. Overall, the agreement between these estimates is excellent.

### Lateral Fenestrations

Lateral fenestrations are proposed as an alternative pathway for ions to enter or exit the channel, distinct from the axial (“apical”) pathway. To determine the extent to which this pathway is followed by ions permeating through the α7-nAChR, the trajectories of ions were tracked as they moved from the bulk solution into (or out of) the channel. Ions moving laterally through ICD cavities (the cytosolic “portals”) were not included in this analysis because their structure remains unsolved.

**Figure 8:**
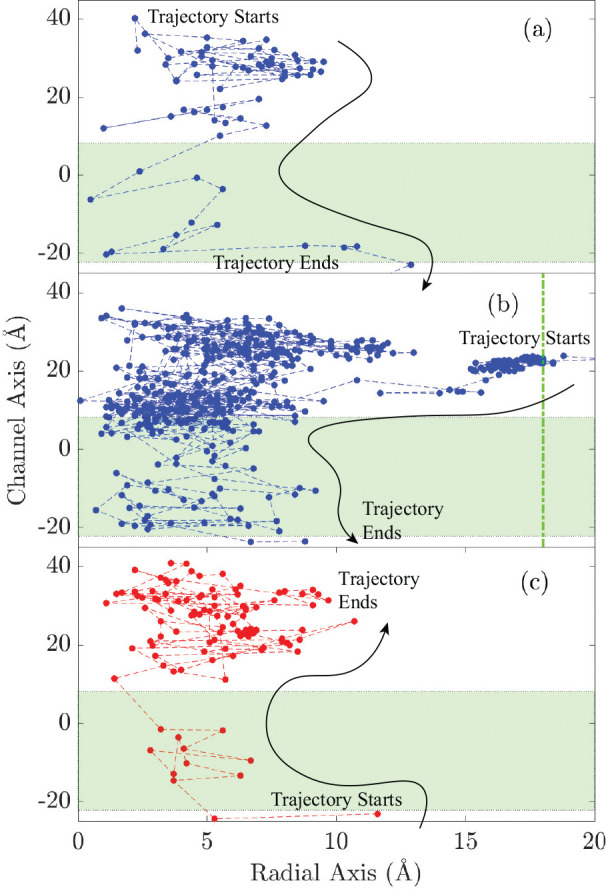
Ion movement: TB event (a), where K^+^ enters the channel axis and passes through TM; (b) K^+^ entering laterally into the ECD and moves through TMD into ICD. The green vertical (dashed-dotted) line shows the threshold to define boundary of a laterally event. (c) Cl^−^ entering ICD crossing TMD and out into the ECD axially. Zero is the position of Val 251 on the vertical axis and shaded region is the TMD; ECD and ICD are in positive and negative vertical regions of the plot, respectively.

Fig. 8 shows the analysis of two ${TB}_{K^{+}}$ permeation events in blue and one ${BT}_{{Cl}^{-}}$ permeation event in red. These are partial tracks of the ions passing through the TMD taken from one of the MD simulations (indexed 6.a of table 1). The position of ions on the plots are marked every 100 ps, and dashed lines join them in the order in which they occurred. The middle plot shows a K^+^ permeation event that started with an excursion of the ion into the channel through the “walls” of the ECD. Visual inspection of the trajectory shows that the ion crosses the channel walls in-between two subunits, passing through the opening between the β8−β9 and β1−β2 loops. Potassium ions taking this pathway always amount to a positive event; in other words, we did not observe any K^+^ exit the channel through these lateral openings in the ECD. Also, we did not observe any chloride ion take this pathway in either direction, possibly due to lack of chloride events.

The rate of ion permeation through these lateral fenestrations is ~1.4 events in 1 μs. In the 10 MD runs, worth 3.6 μs at $V_{e}=-102\;mV$, there were a total of 5 permeation events in which the ion entered the channel through these lateral entryways. Thus, this pathway was followed by 0.6% of ${TB}_{K^{+}}$ events (of 794 total ${TB}_{K^{+}}$ events) and 0.4% of total events (of 1,089 total events in either direction). In marked contrast, in the anion-selective Cys-loop receptor α1-GlyR, MD simulations showed a much higher contribution of lateral pathways amounting to 96% of total events (717 lateral events of total 748 events in both directions) for Cl^−^.^52^ Despite its low probability and longer time to cross the lateral boundary, there exist clear openings between the subunits of the receptor that allow ions to enter the channel.

## Conclusion

We conducted MD simulations on three atomic models of the α7-nAChR believed to represent the open-channel conformation (PDB IDs: 7KOX, 7EKT, and 8V80). Despite being resolved under similar experimental conditions, only one model—7KOX was found to be conductive in our simulations. The other two structures, 7EKT and 8V80, exhibited persistent hydrophobic gating, which likely impeded ion permeation.

Consistent with previous computational studies, the 7KOX structure supported ion conduction. Simulations performed across a range of membrane voltages revealed Ohmic behavior, with conductance increasing under positive voltage relative to negative, in line with earlier studies^10,11^ with sodium ions. The single-channel conductance was computed based on net permeation events of K^+^ and Cl^−^ ions in both extracellular-to-intracellular and intracellular-to-extracellular directions. The combined K^+^/Cl^−^ conductance was calculated to be 219±22 pS, closely matching our experimental estimate of 193±2 pS within error margins.

We carried out multiple simulations ranging from 200 ns to 1 μs and totaling in 3.6 μs. When segmented into 200 ns intervals, the conductance ranged from 79 pS to 399 pS, stressing the importance of conducting multiple simulations for accurate conductance calculations. This variability was higher in the intracellular-to-extracellular direction of K^+^ conduction events, suggesting an asymmetry in conduction behavior. We note, however, that we have not included the full intracellular part of the protein, which was not revealed in the PDB structure, and may well have an effect on ion conduction.

To investigate potential structural determinants of conductance variability, we analysed several structural features—including the positions of charged side chains along the pore axis, pore-radius profiles, electrostatic potentials, ion-density distributions, and hydration patterns of K^+^ ions (including RMSD variation, PCA, and tSNE analysis between different copies). However, no clear correlation between these features and conductance was identified.

Ion permeation appeared to be a Poisson process. Notably, there was a discernible lag between consecutive permeation events, which we attributed to electrostatic repulsion among cations during initial channel entry. We modelled this behaviour using a double-Poisson process, comprising two linked Poisson distributions to represent these sequential processes: one for the lag time and another for the inter-event waiting times.

Interestingly, the conductive model (7KOX) also allowed ions to enter laterally through extracellular-domain (ECD) fenestrations. However, only a small fraction (0.4%) of the total ion crossings occurred via this slower lateral pathway.

While our simulations offer a detailed mechanistic perspective on ion conduction through the α7-nAChR, they do not resolve the mechanisms underlying the discrete subconductance levels observed experimentally. The absence of the complete intracellular domain in our structural model may omit key electrostatic or structural contributions that affect conductance states. Moreover, the lack of modulatory ligands in our simulations — used experimentally to prolong open states—could further limit the correspondence between computed and electrophysiologically obtained values. Although prior work has suggested that desensitized states may retain residual conductance, our simulations focus exclusively on the open state, and thus do not address this possibility. Future studies incorporating full-length receptor models, modulatory compounds, and alternative conformations will be necessary to fully resolve the structural basis of α7-AChR subconductance behaviour.

## Supplementary Material

Supplement 1

## Figures and Tables

**Figure 1:**
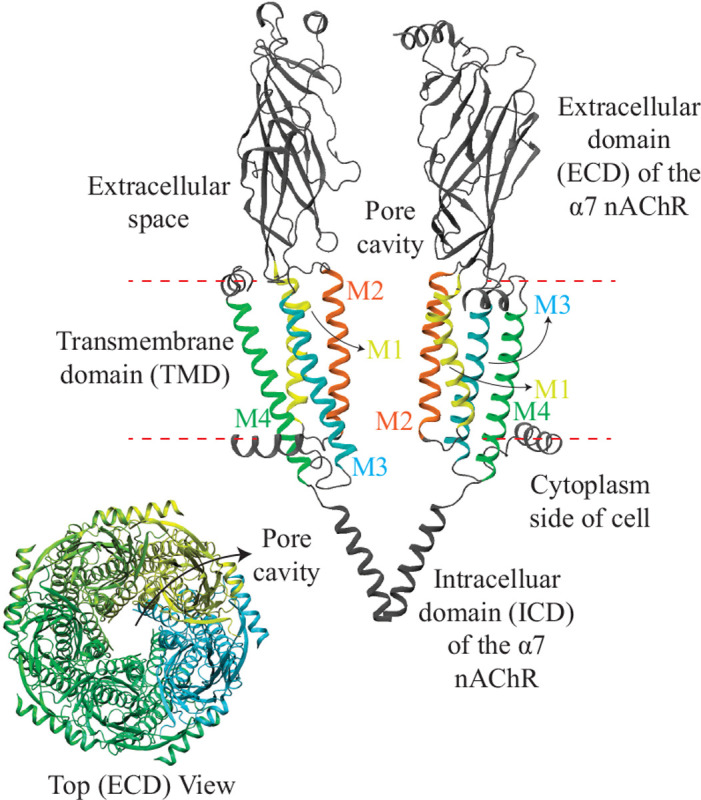
Upper right: Two non-adjacent subunits of the α7-nAChR structure in PDB ID 7KOX. The TMD, embedded in the lipid bilayer (red dashed lines), is composed of M1 to M4 α-helices, coloured differently. The M2 α-helix in orange is exposed to the pore of the channel. Lower left: Top view showing all five α7 subunits of the receptor, each coloured differently. The pore cavity through which ions move is labelled.

**Table 1:** Summary of events at $V_{e}=-102\;mV$. Simulations ran longer are broken in intervals of 200 ns. The total run time of these simulations is 3.6 μs. The first four columns from the left, after indices of the runs, are TB and BT event counts from all simulations for K^+^ and Cl^−^ ions as marked. The net events, ${TB}_{K^{+}}+{BT}_{{Cl}^{-}}-{BT}_{K^{+}}-{TB}_{{Cl}^{-}}$, are used to calculate the conductance, in pS, which are given in the last 2 column. The average of quantities in each column is given in the last row of the table with standard error.

Index	${TB}_{K^{+}}$	${BT}_{K^{+}}$	${TB}_{{Cl}^{-}}$	${BT}_{{Cl}^{-}}$	Net Events	Conductance [pS]

1	50	17	0	0	33	260
2	40	17	0	0	23	181
3	32	20	0	0	12	94
4	39	27	0	0	12	94
5	31	21	0	0	10	79

6.a	45	12	0	1	34	264
6.b	45	16	0	0	29	225

7.a	32	15	0	1	18	142
7.b	28	6	0	0	22	174

8.a	40	23	0	0	17	134
8.b	62	16	0	0	46	362

9.a	51	18	0	0	33	260
9.b	43	13	0	1	31	244

10.a	47	16	0	0	31	242
10.b	39	10	0	1	30	234
10.c	62	13	1	1	49	383
10.d	65	14	0	0	51	399
10.e	43	21	0	0	22	171

Average						

-	44 ± 2	16 ± 1	0±0	0 ± 0	28 ± 3	219 ± 22
