# Supplementary material for: Characterizing the Ion-Conductive State of the α7-Nicotinic Acetylcholine Receptor via Single-Channel Measurements and Molecular Dynamics Simulations
Source: bioRxiv. 2025 Aug 19:2025.08.15.670429. Preprint. [Version 1] doi: 10.1101/2025.08.15.670429 (PMC12393385; doi:10.1101/2025.08.15.670429)
Supplement: Supplement 1 [file NIHPP2025.08.15.670429v1-supplement-1.pdf]

bioRxiv preprint doi: https://doi.org/10.1101/2025.08.15.670429; this version posted August 19, 2025. The copyright holder for this preprint (which was not certified by peer review) is the author/funder. This article is a US Government work. It is not subject to copyright under 17 USC 105 and is also made available for use under a CC0 license.

Supporting Information Available

Table I: Summary of the 6 step equilibration protocol used before any production run. The voltage difference is 0 mV (no applied EF) during these steps. Th 3rd column is applicable for PDB IDs 7KOX and 8V80.

Ensemble	Duration (ns)	GLU:44/Ca^{+2} Restraint (kcal/mol/Å^2)	Position Restraint (Protein) (kcal/mol/Å^2)	Position Restraint (Lipid) (kcal/mol/Å^2)	Dihedral Restraint (Lipid) (kcal/mol/rad^2)
NVT	0.025	50	10	2.5	250
NVT	0.025	50	5	2.5	100
NPT	0.05	50	2.5	1	50
NVT	0.1	50	1	0.5	50
NVT	0.1	50	0.5	0.1	25
NPT	2	50	none	none	none

Table II: Summary of initial production runs.

PDB ID	Copies	Software	Ensemble	Replica Duration (ns)	Voltage (mV)
7KOX	3	AMBER	NVT	200	−102
7EKT	3	OPENMM	NPT	200	−102
8V80	3	AMBER	NVT	200	−102

Table III: Summary of simulation setup details.

PDB ID	Box Size (x,y,z) (Å)	Lipid Count (POPCs) Upper/Lower	Disulfide bonds Cysteine IDs
7KOX	124×124×172	120/116	141-127 & 189-190
7EKT	124×124×197	120/125	150-164 & 212-213
8V80	128×128×192	118/131	141-127 & 189-190

Table IV: Summary of software used for each realization and simulation times at voltage $V_e = -102$ mV.

Copies	Software	Equilibration (Duration (ns))	Production (Duration (ns))
4	OPENMM	NPT (5)	NPT (400)
1	AMBER	NPT (10)	NVT (1000)
5	AMBER	NPT (10)	NVT (200)

bioRxiv preprint doi: https://doi.org/10.1101/2025.08.15.670429; this version posted August 19, 2025. The copyright holder for this preprint (which was not certified by peer review) is the author/funder. This article is a US Government work. It is not subject to copyright under 17 USC 105 and is also made available for use under a CC0 license.

Table V: Summary of production runs (PDB ID 7KOX) at different voltages. Negative and positive voltages create inward and outward currents, respectively. The current calculated for $V_e = -102$ mV is based on all 10 runs, others are based on 5 runs.

Copies	Software	Ensemble	Replica Duration (ns)	Voltage (mV)	Current (pA)	Conductance (pS)
5	AMBER	NVT	200	-306	-67.6 ± 7.9	220 ± 26
5	AMBER	NVT	200	-204	-42.1 ± 4.5	206 ± 22
10	See table IV for details			-102	-1.8 ± 2.3	175 ± 23
5	AMBER	NVT	200	± 0	1.8 ± 0.3	n/a
5	AMBER	NVT	200	$+102$	36.5 ± 2.5	358 ± 24
5	AMBER	NVT	200	$+204$	82.7 ± 6.6	405 ± 33

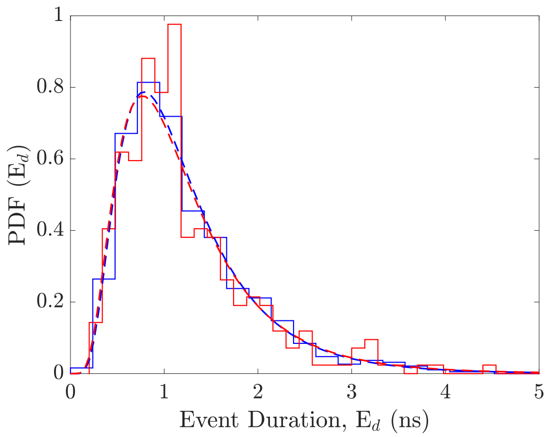

Figure I: Probability density distribution of event duration E_d. This is the duration (in ns) a K^+ ion takes to cross the TMD of the channel, distributions of TB and BT events are coloured blue and red respectively. The dashed lines on the plot are log-normal distribution fitted to the data, with location parameter $\mu = 0.06$ for both distribution, and scaling parameters $\sigma_{TB} = 0.55$ and $\sigma_{BT} = 0.56$.

bioRxiv preprint doi: https://doi.org/10.1101/2025.08.15.670429; this version posted August 19, 2025. The copyright holder for this preprint (which was not certified by peer review) is the author/funder. This article is a US Government work. It is not subject to copyright under 17 USC 105 and is also made available for use under a CC0 license.

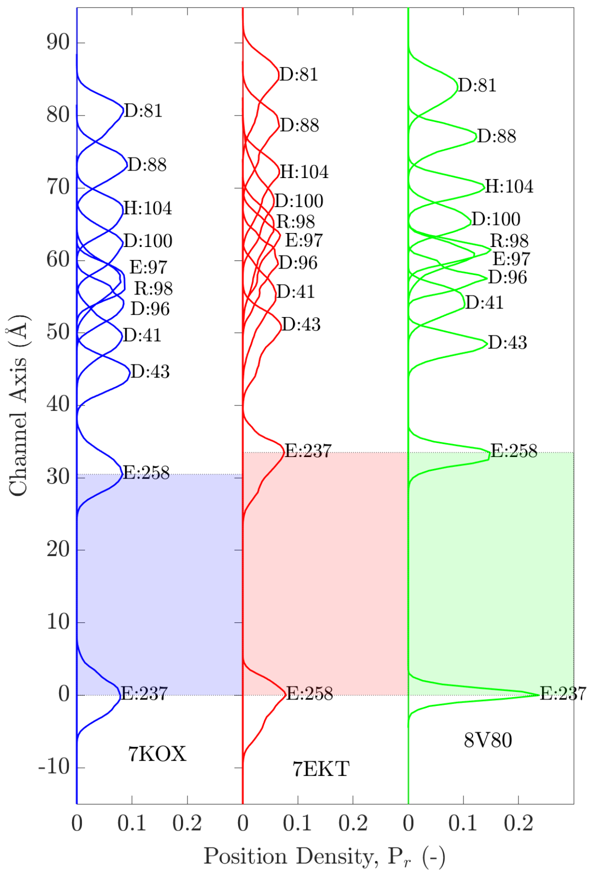

Figure II: The average position of the ionizable residues along the pore of the channels for 3 simulated structures compared. The shaded regions represent TMDs in each case and structures are vertically referenced to Glu 237. Note that the TMD region of 7KET and 8V80 is longer than that of 7KOX along the pore axis.

bioRxiv preprint doi: https://doi.org/10.1101/2025.08.15.670429; this version posted August 19, 2025. The copyright holder for this preprint (which was not certified by peer review) is the author/funder. This article is a US Government work. It is not subject to copyright under 17 USC 105 and is also made available for use under a CC0 license.

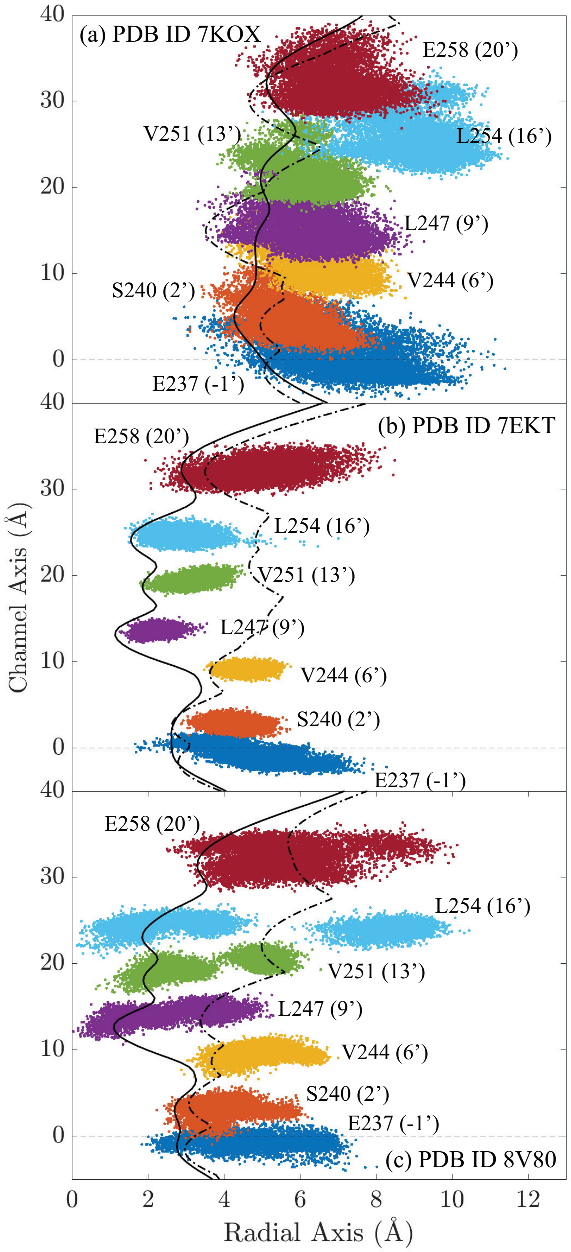

Figure III: Variations during MD simulations in the residues pointing towards the lumen along the M2 α-helix. Each data point is the position of the closest atom to the centre of the pore of each of the 5 subunits. The x-y plane, centred to the middle of the pore, is mapped onto the radial axis and the axis of the channel is referenced to the average position of Glu 237. The results shown here are for 200 ns, and positions are plotted every 100 ps. The black solid and dash-dotted curves are the average HOLE profiles from the simulations and the single HOLE profile calculated from the PDB structure, respectively. The two distinct regions of Leu 254 (also two for Val 251 and Leu 247) of PDB 8V80, for example, are due to the ellipticity of the pore, which is most obvious in simulations of this model. The marked vertical variation in PDB 7KOX is due to the side chains pointing out of the x-y (radial) plane.

bioRxiv preprint doi: https://doi.org/10.1101/2025.08.15.670429; this version posted August 19, 2025. The copyright holder for this preprint (which was not certified by peer review) is the author/funder. This article is a US Government work. It is not subject to copyright under 17 USC 105 and is also made available for use under a CC0 license.

Correlation Analysis between Conductance and Structural Variation

RMSD Variation between Average Structures

From the 10 individual replicas of the conducting system, PBD ID 7KOX, we constructed the average structures of the receptor using CPPTRAJ using the first 200 ns of each run. The average structure represents the average position of each atom from the simulation run, with reference to the initial structure. The temporal average is calculated every 100 ps. Root mean square displacement (RMSD) of the residues which form the inner lining of the pore between structures from different runs is calculated using MDTraj. This shows how the shape of the average pores varies between structures in different runs. The residues included here are, in order from top of the channel (ECD) to the bottom (ICD), as follows: Lys 8, Lys 12, Val 11, Asn 15, Asp 81, Gln 83, Glu 97, Arg 98, His 104, Lys 45, Asn 46, Asp 43, Glu 258, Glu 237, Gly 236, Ser 240, Thr 244, Val 251. These RMSD values were sorted by the number of net events for the duration of the simulation and the variation matrix was computed, Fig. IV. If there was a correlation between net events and average structure, then the lowest RMSD would correspond to the smallest difference between net events. However the matrix has a random pattern, showing no obvious relationship between RMSD values of structures and net events.

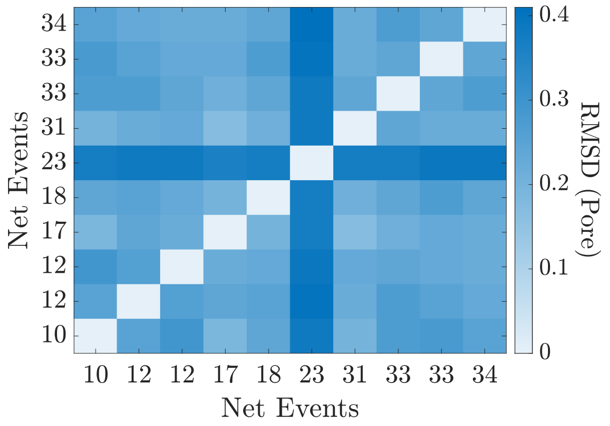

Figure IV: RMSD variation matrix between average structures sorted based on net events from each run at $V_e = -102$ mV.

bioRxiv preprint doi: https://doi.org/10.1101/2025.08.15.670429; this version posted August 19, 2025. The copyright holder for this preprint (which was not certified by peer review) is the author/funder. This article is a US Government work. It is not subject to copyright under 17 USC 105 and is also made available for use under a CC0 license.

PCA and tSNE Analysis

We analyzed the structural states of the α7-nAChR using 120 features of two sets of data, each derived from 10 independent 200 ns simulations (first 200 ns of each replica). These features include pairwise distances between the same residues across all the five chains, with 10 distances per residue. The first data set covers charged residues which face the pore in the ECD and TMDs: Asp 41 (CG atom), Asp 43 (CG atom), Asp 88 (CG atom), Asp 96 (CG atom), Glu 97 (CD atom), Arg 98 (CZ atom), Asp 100 (CG atom), His 104 (CE1 atom), Glu 237 (CD atom), and Glu 258 (CD atom), totalling 100 distance features. We also considered four types of dihedral angles (O-C-CA-CB, C-CA-CB-CG, N-CA-CB-CG, and CA-CB-CG-CD) of Glu 237 on five chains, resulting in additional 20 dihedral angle features in this first data set, composing a total of 120 features. The second data set includes residues within the TMD of the receptor facing the lumen of the channel: Glu 258 (CD atom), Leu 254 (CG atom), Val 251 (CB atom), Leu 247 (CG atom), Val 244 (CB atom), Ser 240 (CB atom) and Glu 237 (CD atom), as shown on Fig. III, resulting in total of 70 distance features. Before applying machine learning algorithms, we normalized all features by subtracting the mean and dividing by one standard deviation. To reduce dimensionality, we first performed Principal Component Analysis (PCA) using the PCA function from the sklearn.decomposition package. Fig. V presents the first and second principal components, with each point representing a state in the configurational space projected onto these components, and each run is coloured according to its net conduction events. No clear correlation was observed between the number of conduction events and configurational states.

Since PCA is a linear transformation and may not capture non-linear relationships, we further applied t-distributed stochastic neighbour embedding (tSNE) for visualization in a 2D space, using the TSNE function from the sklearn.manifold package. The TSNE function was applied only to the features from charged residues, first set of data as detailed before. Instead of using the raw 120-feature data, we input the first 50 PCA components into tSNE to mitigate computational costs and account for tSNE's assumption of local linearity,

bioRxiv preprint doi: https://doi.org/10.1101/2025.08.15.670429; this version posted August 19, 2025. The copyright holder for this preprint (which was not certified by peer review) is the author/funder. This article is a US Government work. It is not subject to copyright under 17 USC 105 and is also made available for use under a CC0 license.

which may not hold in high-dimensional spaces with complex manifolds. The resulting 2D embedding, shown in Fig. VI and coloured by the number of net conduction events, similarly did not reveal any clear correlation between conduction and configuration.

In summary, our PCA and tSNE analyses do not support the hypothesis that variations in conductance arise from differences in the channel's configurational state. Our selected structural features represent only a subset of those necessary to fully characterize the entire protein, yet these selected ones are which directly influence ion crossing through the pore of channel. Expanding the set of features to include more key residues along the conduction pathway may enhance our ability to identify distinct configurational states and further elucidate the relationship between protein structure and conductance.

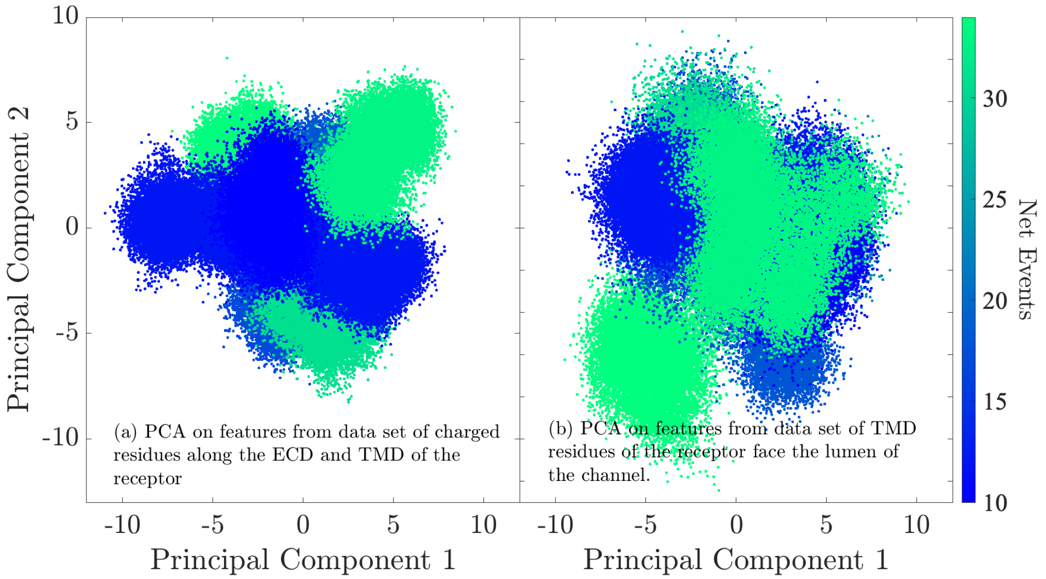

Figure V: First and second principal components derived from Principal Component Analysis (PCA). Left and right panels show results generated from features of the charged residues along the pore and the TMD facing residues respectively. Each dot represents a snapshot sampled at 10 ps intervals, resulting in a total of 200,000 data points from ten independent simulations. The colour of each dot indicates the net conduction event associated with its respective simulation.

bioRxiv preprint doi: https://doi.org/10.1101/2025.08.15.670429; this version posted August 19, 2025. The copyright holder for this preprint (which was not certified by peer review) is the author/funder. This article is a US Government work. It is not subject to copyright under 17 USC 105 and is also made available for use under a CC0 license.

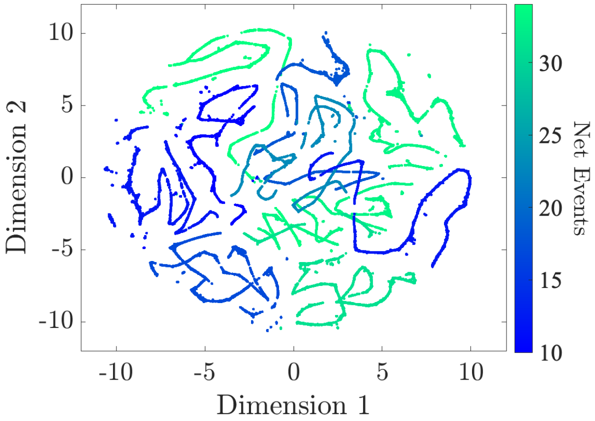

Figure VI: The first and second dimensions derived from t-distributed stochastic neighbour embedding (t-SNE), using the first 50 principal component analysis (PCA) components. Each dot represents a snapshot sampled at 10 ps intervals, resulting in a total of 200,000 data points from ten independent simulations. The colour of each dot indicates the net conduction event associated with its respective simulation.

bioRxiv preprint doi: https://doi.org/10.1101/2025.08.15.670429; this version posted August 19, 2025. The copyright holder for this preprint (which was not certified by peer review) is the author/funder. This article is a US Government work. It is not subject to copyright under 17 USC 105 and is also made available for use under a CC0 license.

Model of Double-Poisson Process

We fitted the distributions of waiting times between events from the simulations to a double-Poisson model. This model comprised of two sequential Poisson distributions, where the output of the first serves as the input of the second distribution. This model assumed events were to be prepared before they conduct subsequently. Starting with the total number of unready events, N_{unready}. Number of events which are ready to conduct, N_{ready}, are define by the lag time before crossing and is governed by the rate of lag, λ_{lag}. Events which are ready and conduct are defined by the conduction rate, λ_{cond}.

The three rate equations of the distributions of these events are

$$\frac{d}{dt}N_{\text{unready}}(t) = -\lambda_{\text{lag}}N_{\text{unready}}, \tag{7a}$$

$$\frac{d}{dt}N_{\text{ready}}(t) = \lambda_{\text{lag}}N_{\text{unready}} - \lambda_{\text{cond}}N_{\text{ready}}, \tag{7b}$$

$$\frac{d}{dt}N_{\text{cond}}(t) = -\lambda_{\text{cond}}N_{\text{ready}}. \tag{7c}$$

A simple integration of the first differential equation gives the distribution of the unready events, N_{unready}, which is an exponential decay with a rate constant λ_{lag}. The solution is:

$$N_{\text{unready}}(t) = N_0 \exp\left[-\lambda_{\text{lag}}t\right]. \tag{8}$$

Plugging this expression in the second differential equation, eq. 7b, gives

$$\frac{d}{dt}N_{\text{ready}}(t) = \lambda_{\text{lag}}N_0 \exp\left[-\lambda_{\text{lag}}t\right] - \lambda_{\text{cond}}N_{\text{ready}}.$$

Reshuffling this and multiplying it with the integration factor $\mu = \exp\left[\lambda_{\text{cond}}t\right]$ gives,

$$\exp\left[\lambda_{\text{cond}}t\right]\frac{d}{dt}N_{\text{ready}}(t) - \exp\left[\lambda_{\text{cond}}t\right]\lambda_{\text{cond}}N_{\text{ready}} = \exp\left[\lambda_{\text{cond}}t\right]\lambda_{\text{lag}}N_0 \exp\left[-\lambda_{\text{lag}}t\right].$$

bioRxiv preprint doi: https://doi.org/10.1101/2025.08.15.670429; this version posted August 19, 2025. The copyright holder for this preprint (which was not certified by peer review) is the author/funder. This article is a US Government work. It is not subject to copyright under 17 USC 105 and is also made available for use under a CC0 license.

Following the Leibniz rule, the expression becomes

$$\frac{d}{dt}\left(\exp\left[\lambda_{\text{cond}}t\right]N_{\text{ready}}\left(t\right)\right)=\exp\left[\lambda_{\text{cond}}t\right]\lambda_{\text{lag}}N_0\exp\left[-\lambda_{\text{lag}}t\right].$$

Finally, integrating and simplifying this we get a close form solution for the distribution of number of ready events, N_{ready}:

$$\begin{aligned}\int\frac{d}{dt}\left(\exp\left[\lambda_{\text{cond}}t\right]N_{\text{ready}}\left(t\right)\right)dt&=\lambda_{\text{lag}}N_0\int\exp\left[\left(\lambda_{\text{cond}}-\lambda_{\text{lag}}\right)t\right]dt,\\ \exp\left[\lambda_{\text{cond}}t\right]N_{\text{ready}}\left(t\right)&=\frac{\lambda_{\text{lag}}N_0}{\lambda_{\text{cond}}-\lambda_{\text{lag}}}\exp\left[\left(\lambda_{\text{cond}}-\lambda_{\text{lag}}\right)t\right],\\ N_{\text{ready}}\left(t\right)&=\frac{\lambda_{\text{lag}}N_0}{\lambda_{\text{cond}}-\lambda_{\text{lag}}}\left(\exp\left[-\lambda_{\text{lag}}t\right]-\exp\left[-\lambda_{\text{cond}}t\right]\right).\end{aligned}\tag{9}$$

Using this expression in the last differential equation, eq. 7c, and integrating for the number of conducted events, N_{cond}:

$$\begin{aligned}\frac{d}{dt}N_{\text{cond}}\left(t\right)&=-\frac{\lambda_{\text{cond}}\lambda_{\text{lag}}N_0}{\lambda_{\text{cond}}-\lambda_{\text{lag}}}\left(\exp\left[-\lambda_{\text{lag}}t\right]-\exp\left[-\lambda_{\text{cond}}t\right]\right),\\ N_{\text{cond}}\left(t\right)&=-\int\frac{\lambda_{\text{cond}}\lambda_{\text{lag}}N_0}{\lambda_{\text{cond}}-\lambda_{\text{lag}}}\left(\exp\left[-\lambda_{\text{lag}}t\right]-\exp\left[-\lambda_{\text{cond}}t\right]\right)dt.\end{aligned}$$

The final solution is

$$N_{\text{cond}}\left(t\right)=\frac{N_0}{\lambda_{\text{cond}}-\lambda_{\text{lag}}}\left(\lambda_{\text{cond}}\exp\left[-\lambda_{\text{lag}}t\right]-\lambda_{\text{lag}}\exp\left[-\lambda_{\text{cond}}t\right]\right).\tag{10}$$

This gives the distribution of events conducted in time from the total number of events, N_0, in the start. Hence, the cumulative distribution function, $F_{\text{events}}\left(t\right)$, is

$$F_{\text{events}}\left(t\right)=N_0-\frac{N_0}{\lambda_{\text{cond}}-\lambda_{\text{lag}}}\left(\exp\left[-\lambda_{\text{lag}}t\right]-\exp\left[-\lambda_{\text{cond}}t\right]\right),\tag{11}$$

bioRxiv preprint doi: https://doi.org/10.1101/2025.08.15.670429; this version posted August 19, 2025. The copyright holder for this preprint (which was not certified by peer review) is the author/funder. This article is a US Government work. It is not subject to copyright under 17 USC 105 and is also made available for use under a CC0 license.

and the probability density function, $f_{\text{events}}(t)$, is

$$f_{\text{events}}(t) = \frac{d}{dt} F_{\text{events}}(t), \\ = -\frac{\lambda_{\text{cond}}\lambda_{\text{lag}}N_0}{\lambda_{\text{cond}} - \lambda_{\text{lag}}} \left(\exp\left[-\lambda_{\text{lag}}t\right] - \exp\left[-\lambda_{\text{cond}}t\right]\right). \quad (12)$$

Number of ready and conducted events were defined to be zero at the start time, $t_i = 0$, and probability is normalized to unity, which implies $N_0 = 1$.

Evidently, distributions given in above equations are defined by two independent rate parameters. The time lag before an event occurs is defined by the first parameter, λ_{lag}, and rate of conduction is defined by the λ_{cond}. These rate parameters of probability density function of the number of conducted events were optimized to the waiting time distribution of the simulated results. Plots of these three distributions with rate parameters as used for the TB and BT waiting distributions from the simulations are shown in Fig. VII.

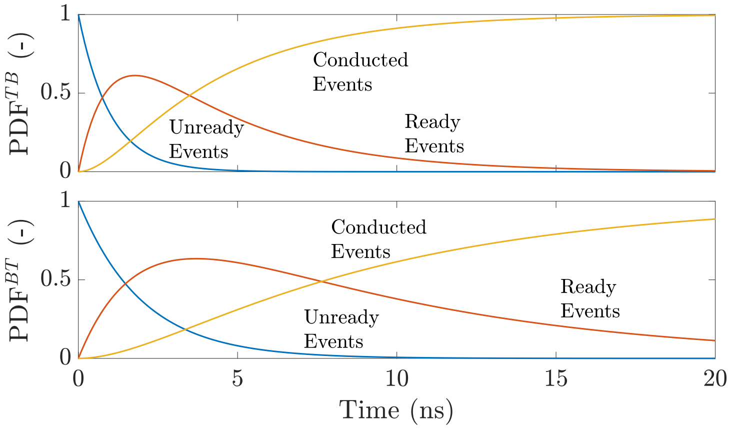

Figure VII: Probability of events over time, at each of the two steps of the double-Poisson model. The top and bottom panels show TB and BT events respectively. The blue plots represent the probability of the total number of unready events (eq. 8). This probability decreases over time as events prepare to conduct after waiting a period, defined as the lag time. The red plots show the probability of events (eq. 9) that have completed the waiting period and are ready to conduct. This distribution increases until events begin to conduct, reflecting the delay introduced by the lag time. Finally, the yellow plots (eq. 10) depict the probability of events that have conducted.
